# Supplementary material for: Burnout among medical students of a medical college in Kathmandu; A cross-sectional study
Source: PLoS One. 2021 Jun 24;16(6):e0253808. doi: 10.1371/journal.pone.0253808 (PMC8224915; doi:10.1371/journal.pone.0253808)
Supplement: S1 File — (PDF) [file pone.0253808.s001.pdf]

Nepalese Army Institute of Health Sciences (NAIHS)
Sanobharyang, Bhandarkhal, Kathmandu
Research Proposal Form – 2077 (2020)

Date: 2020/11/29

Proposed Title of the Research: Burnout among medical students of a medical college in Kathmandu; A Cross Sectional Study

Name of Investigators:

No	Name In full	Highest Degree	Department	Role in Research
1	Nagendra Katuwal	MD (Neuro-psychiatry)	Dept. of Psychiatry	Principal Investigator
2	Ayush Tamang	MBBS 3rd year student		Co-investigator
3	Agrima Paudel	MBBS 3rd year student		Co-investigator
4	Anu Gautam	MBBS 3rd year student		Co-investigator
5	Muna Sharma	MBBS 3rd year student		Co-investigator
6	Ujwal Bhusal	MBBS 3rd year student		Co-investigator

Place for Research: Nepalese Army Institute of Health Sciences - College of Medicine

Affiliations: Shree Birendra Hospital, Nepalese Army Institute of Health and Sciences

Funding Source : None

Technical Staff involved: No

Office Staff: No

Burnout among medical students of a medical college in Kathmandu; A Cross Sectional Study

Research Proposal

1. Introduction & Justification

Academic burnout can be defined as a phenomenon that is characterized by feelings of (emotional, physical, and cognitive) exhaustion due to the demands of studying and an attitude of withdrawal and detachment from one's studies [1].

Numerous studies have shown that medical students experience high levels of burnout due to a highly stressful environment, competitiveness, excessive workload, sleep deprivation, peer pressure, and many other personal, curricular, institutional, and affective factors [2]. Studies on medical students have indicated the development of stress and burnout in the preclinical medical education and its progression into clinical years [3] [4]. Burnout in medical students has been associated with depression, sleep deprivation, thoughts of dropping out, suicidal ideation and substance abuse [5] [6] [2] [7]. It can also mitigate cognitive capabilities such as memory, interpretation of information, and skill acquisition [8].

Furthermore, burnout of medical students can also lead to burnout after they become a doctor [9] which may result in unprofessionalism, poor quality of patient care, medical errors, suicidal ideation and attrition, and be a factor in substance abuse and relationship difficulties [10] [11]

Many studies have found that the prevalence of burnout among medical students, residents and physicians is as much as 50% in the US [12] [13]. Around the globe prevalence rates for medical students burnout ranges from 7.0% to 75.2%, depending on country-specific factors, applied instruments, cutoff-criteria for burnout symptomatology [14]. However, burnout has not been adequately explored in the context of Nepal.

Therefore, we plan to conduct a cross sectional study with an aim to find out the prevalence of burnout among medical students of Nepalese Army Institute of Health Sciences-College of Medicine (NAIHS-COM).

2. Research objectives:

- To measure the prevalence of burnout among medical students of a medical college.
- To compare the prevalence of burnout among students of different academic years, age and gender.

3. Methodology:

3.1 Study design

Cross-sectional study

3.2 Sampling method with sample size

Stratified sampling technique using students' class year as a stratum followed by simple random sampling method using random number generator software.

Sample size was calculated as:

$N = Z^2pqN/(E^2(N-1) + Z^2pq)$

$= 228$

Where,

n = Sample size

Z = 1.96 at 95% CI

p = 0.49 (taken 0.49 based on 48.8% prevalence of burnout in a similar Nepalese study) [15]

q = 1-p

N = sampling population = 560

E = marginal error = 5%

Therefore, the calculated sample size is 228 and considering 5% of non-response rate the sample size will be 239.

3.3 Study sites & its justification

Nepalese Army Institute of Health Sciences, College of Medicine(NAIHS-COM)

1. Medical students are more prone to burnout than the general population of the same age group.
2. The research on this topic hasn't been conducted here before.
3. Convenient to collect data .

3.4 Study population

First to fifth year medical students of NAIHS-COM.

3.5 Study Period

4 months after the resumption of college

3.6 Sampling Unit

Individual

3.7 Data collections techniques

Oldenburg Burnout Inventory(OLBI) based Self Administered Questionnaire

3.8 Data collections tool

Oldenburg Burnout Inventory-Student Version (OLBI-S)

3.9 Potential biases

1. Selection bias
2. Information bias

3.10 Conceptual framework

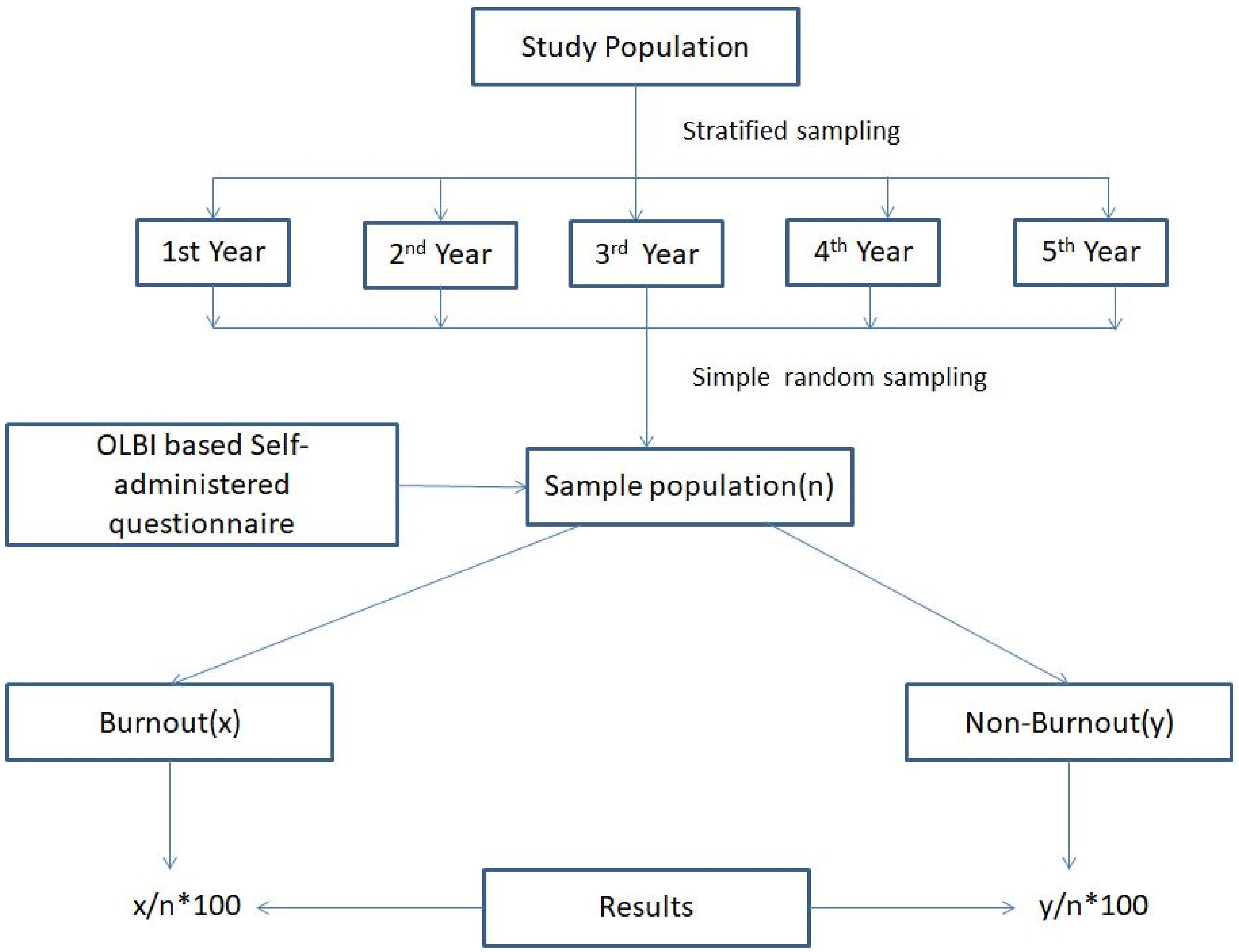

4. Intervention

None

5. Expected results

In accordance with the previous studies [15][16], we expect the prevalence of burnout to be around 40-50%.

6. Application and uses:

1. Burnout in students
   - has a negative impact on their personal life and health.
   - may lead to a negative attitude towards their study, decline in academic performance and even dropout.
   - may continue into their professional life when they become doctor and affect patient care.
2. Our research would contribute to raising awareness about burnout among the medical community.
3. Our findings would reinforce the need to establish preventive measures at an individual as well as at an institutional level.

7. How informed consent is obtained and Ethical consideration (Attach informed consent form if available)

The participation will be entirely voluntary and relevant information regarding our study will be provided to the participants. A letter of consent will be attached at the beginning of our questionnaire.

8. Limitation of study

1. Being a cross-sectional study, associations and causality cannot be ascertained.

2. As the study is conducted in a single medical school, this may not be representative of the national outcomes of burnout in medical students.

3. The choice of response and the degree of honesty in disclosing one's problems might be affected by the level of stigma in our setting.

9. References (Vancouver)

1. Reis, D., Xanthopoulou, D., & Tsaousis, I. (2015). Measuring job and academic burnout with the Oldenburg Burnout Inventory (OLBI): Factorial invariance across samples and countries. Burnout Research, 2(1), 8-18. doi:10.1016/j.burn.2014.11.001

2. Pacheco, J. P., Giacomin, H. T., Tam, W. W., Ribeiro, T. B., Arab, C., Bezerra, I. M., & Pinasco, G. C. (2017). Mental health problems among medical students in Brazil: A systematic review and meta-analysis. Revista Brasileira De Psiquiatria, 39(4), 369-378. doi:10.1590/1516-4446-2017-2223

3. K. Ashkar, M. Romani, U. Musharrafieh, M. Chaaya. Prevalence of burnout syndrome among medical residents: experience of a developing country Postgrad Med J, 86 (2010), pp. 266-271

4 . Fares, J., Saadeddin, Z., Tabosh, H. A., Aridi, H., Mouhayyar, C. E., Koleilat, M. K., . . . Asmar, K. E. (2015). Extracurricular activities associated with stress and burnout in preclinical medical students. Journal of Epidemiology and Global Health, 6(3), 177. doi:10.1016/j.jegh.2015.10.003

5. Youssef, F. F. (2016). Medical Student Stress, Burnout and Depression in Trinidad and Tobago. Academic Psychiatry, 40(1), 69-75. doi:10.1007/s40596-015-0468-9

6. Mazurkiewicz, R., Korenstein, D., Fallar, R., & Ripp, J. (2012). The prevalence and correlations of medical student burnout in the pre-clinical years: A cross-sectional study. Psychology, Health & Medicine, 17(2), 188-195. doi:10.1080/13548506.2011.597770

7. Dyrbye, L., & Shanafelt, T. (2015). A narrative review on burnout experienced by medical students and residents. Medical Education, 50(1), 132-149. doi:10.1111/medu.12927

8. Miranda-Ackerman, R. C., Barbosa-Camacho, F. J., Sander-Möller, M. J., Buenrostro-Jiménez, A. D., Mares-País, R., Cortes-Flores, A. O., . . . González-Ojeda, A. (2019). Burnout syndrome prevalence during internship in public and private hospitals: A survey study in Mexico. Medical Education Online, 24(1), 1593785. doi:10.1080/10872981.2019.1593785

9. Dyrbye, L. N., Thomas, M. R., Huntington, J. L., Lawson, K. L., Novotny, P. J., Sloan, J. A., & Shanafelt, T. D. (2006). Personal Life Events and Medical Student Burnout: A Multicenter Study. Academic Medicine, 81(4), 374-384. doi:10.1097/00001888-200604000-00010

10. Dyrbye, L. N., West, C. P., Satele, D., Boone, S., Tan, L., Sloan, J., & Shanafelt, T. D. (2014). Burnout Among U.S. Medical Students, Residents, and Early Career Physicians Relative to the General U.S. Population. Academic Medicine, 89(3), 443-451. doi:10.1097/acm.0000000000000134

11. Dyrbye, L., & Shanafelt, T. (2015). A narrative review on burnout experienced by medical students and residents. Medical Education, 50(1), 132-149. doi:10.1111/medu.12927

12. Chang, E., Eddins-Folensbee, F., & Coverdale, J. (2012). Survey of the Prevalence of Burnout, Stress, Depression, and the Use of Supports by Medical Students at One School. Academic Psychiatry, 36(3), 177. doi:10.1176/appi.ap.11040079

13. Shanafelt TD, Boone S, Litjen T, et al. Burnout and satisfaction with work-life balance among US physicians relative to the general US population. Arch Intern Med. 2012; 172:13771385.

14. Erschens R, Keifenheim KE, Herrmann-Werner A, et al. Professional burnout among medical students: Systematic literature review and meta-analysis. Med Teach. 2019;41(2):172-183. doi:10.1080/0142159X.2018.1457213

15. Pokhrel NB, Khadayat R, Tulachan P. Depression, anxiety, and burnout among medical students and residents of a medical school in Nepal: a cross-sectional study. BMC Psychiatry. 2020;20(1):298. Published 2020 Jun 15. doi:10.1186/s12888-020-02645-6

16. Dyrbye, L. N., Thomas, M. R., Huntington, J. L., Lawson, K. L., Novotny, P. J., Sloan, J. A., & Shanafelt, T. D. (2006). Personal life events and medical student burnout: a multicenter study. Academic medicine : journal of the Association of American Medical Colleges,81(4),374–384. https://doi.org/10.1097/00001888-200604000-00010

10. Questionnaire: Attached

11. C.V. of principal investigator: Attached

Signature:

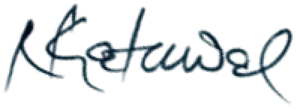

Dr. Nagendra Katuwal

Principal investigator
