# Supplementary material for: Burnout among medical students of a medical college in Kathmandu; A cross-sectional study
Source: PLoS One. 2021 Jun 24;16(6):e0253808. doi: 10.1371/journal.pone.0253808 (PMC8224915; doi:10.1371/journal.pone.0253808)
Supplement: S2 File — (PDF) [file pone.0253808.s002.pdf]

Burnout among medical students of a medical college in Kathmandu; a DCSS

Random number: ________; **Class Year**: ______; **Age** (completed yrs): _______ ; **Gender**: ______

Please read the following questions carefully and tick the most appropriate answer:

S.no.	Question	Strongly agree	Agree	Disagree	Strongly disagree
1	I always find new and interesting aspects in my studies.				
2	There are days when I feel tired before I arrive in class or start studying.				
3	I can usually manage my study-related workload well.				
4	Over time, one can become disconnected from this type of study.				
5	I find my studies to be a positive challenge.				
6	After a class or after studying, I tend to need more time than in the past in order to relax and feel better.				
7	I can tolerate the pressure of my studies very well.				
8	Lately, I tend to think less about my academic tasks and do them almost mechanically.				
9	I feel more and more engaged in my studies.				
10	While studying, I often feel emotionally drained.				
11	After a class or after studying, I have enough energy for my leisure activities.				
12	It happens more and more often that I talk about my studies in a negative way.				
13	This is the only field of study that I can imagine myself doing.				
14	After a class or after studying, I usually feel worn out and weary.				
15	When I study, I usually feel energized.				
16	Sometimes I feel sickened by my studies.				
