# Supplementary material for: Burnout among medical students of a medical college in Kathmandu; A cross-sectional study
Source: PLoS One. 2021 Jun 24;16(6):e0253808. doi: 10.1371/journal.pone.0253808 (PMC8224915; doi:10.1371/journal.pone.0253808)
Supplement: Attachment — Submitted filename: PONE-D-21-09730_reviewer_RKY.pdf [file pone.0253808.s005.pdf]

PLOS ONE

Burnout among medical students of a medical college in Kathmandu; A cross sectional study

--Manuscript Draft--

**Manuscript Number:**	PONE-D-21-09730
**Article Type:**	Research Article
**Full Title:**	Burnout among medical students of a medical college in Kathmandu; A cross sectional study
**Short Title:**	Burnout among medical students; a cross sectional study
**Corresponding Author:**	Dhan Bahadur Shrestha, MBBS<br>Mangalbare Hospital<br>Urlabari, 1 NEPAL
**Keywords:**	Burnout, Professional; Education, Medical; Nepal; Students, Medical
**Abstract:**	Background<br><br>Medical students are more prone to burnout than the general population and students of other faculties due to the demanding nature of medical education with limited time and resources. Burnout has a negative impact on the academics and personal life of the students which can continue into their professional life and ultimately hamper patient care. The purpose of this study is to determine prevalence of burden among medical students of a medical college and find its association with age, gender, and year of study. Materials and Methods<br><br>This cross sectional study was conducted among medical students of a medical college in Kathmandu, Nepal from 14 January to 7 March 2021. Stratified sampling followed by a simple random sampling technique was employed to select study participants. Data was collected through a self-administered questionnaire using Oldenburg Burnout Inventory adapted for students (OLBI-S) and analyzed in STATA version 15.Results<br><br>The prevalence of burnout was found out to be 65.9%. And of the remaining 12.7% were exhausted, 11.4% were disengaged and 10.0% were neither exhausted nor disengaged. The burnout had no significant association with age in years, gender and year of study.Conclusions<br><br>This study shows an alarming prevalence of burnout in almost two-thirds of medical students. These results indicate the necessity of employing effective strategies by relevant authorities for the mental well-being of future physicians. Further multicenter prospective studies are required for a better understanding of the prevalence and associated factors of burnout.
**Order of Authors:**	Dhan Bahadur Shrestha, MBBS
	Nagendra Katuwal
	Ayush Tamang
	Agrima Paudel
	Anu Gautam
	Muna Sharma
	Ujwal Bhusal
	Pravash Budhathoki
**Additional Information:**	
**Question**	**Response**
**Financial Disclosure**	The author(s) received no specific funding for this work.

Powered by Editorial Manager® and ProduXion Manager® from Aries Systems Corporation

Enter a financial disclosure statement that describes the sources of funding for the work included in this submission. Review the submission guidelines for detailed requirements. View published research articles from *PLOS ONE* for specific examples.<br><br>This statement is required for submission and **will appear in the published article** if the submission is accepted. Please make sure it is accurate.<br><br>**Unfunded studies**<br>Enter: *The author(s) received no specific funding for this work.*<br><br>**Funded studies**<br>Enter a statement with the following details:<br>• Initials of the authors who received each award<br>• Grant numbers awarded to each author<br>• The full name of each funder<br>• URL of each funder website<br>• Did the sponsors or funders play any role in the study design, data collection and analysis, decision to publish, or preparation of the manuscript?<br>• **NO** - Include this sentence at the end of your statement: *The funders had no role in study design, data collection and analysis, decision to publish, or preparation of the manuscript.*<br>• **YES** - Specify the role(s) played.<br><br>* typeset	
**Competing Interests**<br><br>Use the instructions below to enter a competing interest statement for this submission. On behalf of all authors, disclose any competing interests that could be perceived to bias this work—acknowledging all financial support and any other relevant financial or non-financial competing interests.<br><br>This statement is required for submission and **will appear in the published article** if	The authors have declared that no competing interests exist.

the submission is accepted. Please make sure it is accurate and that any funding sources listed in your Funding Information later in the submission form are also declared in your Financial Disclosure statement.<br><br>View published research articles from *PLOS ONE* for specific examples.<br><br>**NO authors have competing interests**<br><br>Enter: *The authors have declared that no competing interests exist.*<br><br>**Authors with competing interests**<br><br>Enter competing interest details beginning with this statement:<br><br>*I have read the journal's policy and the authors of this manuscript have the following competing interests: [insert competing interests here]*<br><br>* typeset	
**Ethics Statement**<br><br>Enter an ethics statement for this submission. This statement is required if the study involved:<br><br>• Human participants<br>• Human specimens or tissue<br>• Vertebrate animals or cephalopods<br>• Vertebrate embryos or tissues<br>• Field research<br><br>Write "N/A" if the submission does not require an ethics statement.<br><br>General guidance is provided below. Consult the submission guidelines for detailed instructions. **Make sure that all information entered here is included in the Methods section of the manuscript.**	Ethical clearance was obtained from the Institutional Review Committee (IRC) of NAIHS before collecting data from participants (Ref no: 365). All the respondents were informed about the aim and objectives of the study by including the consent form in the questionnaire itself. The participants were aware that their participation was voluntary. Confidentiality of the participants was ensured.

**Format for specific study types**<br><br>**Human Subject Research (involving human participants and/or tissue)**<br>• Give the name of the institutional review board or ethics committee that approved the study<br>• Include the approval number and/or a statement indicating approval of this research<br>• Indicate the form of consent obtained (written/oral) or the reason that consent was not obtained (e.g. the data were analyzed anonymously)<br><br>**Animal Research (involving vertebrate animals, embryos or tissues)**<br>• Provide the name of the Institutional Animal Care and Use Committee (IACUC) or other relevant ethics board that reviewed the study protocol, and indicate whether they approved this research or granted a formal waiver of ethical approval<br>• Include an approval number if one was obtained<br>• If the study involved *non-human primates*, add *additional details* about animal welfare and steps taken to ameliorate suffering<br>• If anesthesia, euthanasia, or any kind of animal sacrifice is part of the study, include briefly which substances and/or methods were applied<br><br>**Field Research**<br><br>Include the following details if this study involves the collection of plant, animal, or other materials from a natural setting:<br>• Field permit number<br>• Name of the institution or relevant body that granted permission	
**Data Availability**<br><br>Authors are required to make all data underlying the findings described fully available, without restriction, and from the time of publication. PLOS allows rare exceptions to address legal and ethical concerns. See the PLOS Data Policy and FAQ for detailed information.	Yes - all data are fully available without restriction

A Data Availability Statement describing where the data can be found is required at submission. Your answers to this question constitute the Data Availability Statement and **will be published in the article**, if accepted.<br><br>**Important:** Stating 'data available on request from the author' is not sufficient. If your data are only available upon request, select 'No' for the first question and explain your exceptional situation in the text box.<br><br>Do the authors confirm that all data underlying the findings described in their manuscript are fully available without restriction?	
**Describe where the data may be found in full sentences. If you are copying our sample text, replace any instances of XXX with the appropriate details.**<br><br>• If the data are **held or will be held in a public repository**, include URLs, accession numbers or DOIs. If this information will only be available after acceptance, indicate this by ticking the box below. For example: *All XXX files are available from the XXX database (accession number(s) XXX, XXX.).*<br>• If the data are all contained **within the manuscript and/or Supporting Information files**, enter the following: *All relevant data are within the manuscript and its Supporting Information files.*<br>• If neither of these applies but you are able to provide **details of access elsewhere**, with or without limitations, please do so. For example:<br><br>*Data cannot be shared publicly because of [XXX]. Data are available from the XXX Institutional Data Access / Ethics Committee (contact via XXX) for researchers who meet the criteria for access to confidential data.*<br><br>*The data underlying the results presented in the study are available from (include the name of the third party*	All relevant data are within the manuscript and its Supporting Information files.

and contact information or URL).<br>• This text is appropriate if the data are owned by a third party and authors do not have permission to share the data.<br><br>* typeset	
Additional data availability information:	

Manuscript file Click here to access/download;Manuscript;Burnout Manuscript.docx

Title page

Title: Burnout among medical students of a medical college in Kathmandu; A cross sectional study

Authors and affiliation: Dhan Bahadur Shrestha[1*], Nagendra Katuwal[2], Ayush Tamang[3], Agrima Paudel[3], Anu Gautam[3], Muna Sharma[3], Ujwal Bhusal[3], Pravash Budhathoki[4]

[1]Department of Emergency Medicine, Mangalbare Hospital, Morang Province no. 1, Nepal

[2]Department of Psychiatry, Shree Birendra Hospital, Kathmandu, Province no. 3, Nepal

[3]Nepalese Army Institute of Health Sciences, College of Medicine, Kathmandu, Province no. 3, Nepal

[4]Department of Emergency Medicine, Dr. Iwamura Memorial Hospital, Province no. 3, Nepal

Short title: Burnout among medical students; a cross sectional study

Word count (excluding Figure, table and abstract): 2383

Table count: 2

Fig count: 1

***Correspondence**

medhan75@gmail.com

Abstract

Background

Medical students are more prone to burnout than the general population and students of other faculties due to the demanding nature of medical education with limited time and resources. Burnout has a negative impact on the academics and personal life of the students which can continue into their professional life and ultimately hamper patient care. The purpose of this study is to determine prevalence of burden among medical students of a medical college and find its association with age, gender, and year of study.

Materials and Methods

This cross sectional study was conducted among medical students of a medical college in Kathmandu, Nepal from 14 January to 7 March 2021. Stratified sampling followed by a simple random sampling technique was employed to select study participants. Data was collected through a self-administered questionnaire using Oldenburg Burnout Inventory adapted for students (OLBI-S) and analyzed in STATA version 15.

Results

The prevalence of burnout was found out to be 65.9%. And of the remaining 12.7% were exhausted, 11.4% were disengaged and 10.0% were neither exhausted nor disengaged. The burnout had no significant association with age in years, gender and year of study.

Conclusions

This study shows an alarming prevalence of burnout in almost two-thirds of medical students. These results indicate the necessity of employing effective strategies by relevant authorities for the

mental well-being of future physicians. Further multicenter prospective studies are required for a better understanding of the prevalence and associated factors of burnout.

Key-words: Burnout, Professional; Education, Medical; Nepal; Students, Medical

Introduction

Initially, burnout was defined for employees who work with other people, as a syndrome characterized by exhaustion, depersonalization, and reduced professional efficacy [1]. However, over the years, empirical research has shown that burnout is caused due to high job demands and/or lack of job resources [2] regardless of the job that they do [3,4]. Similar to employees, students are also subject to burnout as they have to fulfill certain tasks such as attending class, completing assignments within deadlines, and passing exams [5,6].

In 2007, Demerouti et al. proposed Oldenburg Burnout Inventory (OLBI) considering exhaustion and disengagement to be the two core dimensions of job burnout [7,8]. Reis et al. then adapted OLBI to measure academic burnout (OLBI-S) and defined it as a phenomenon that is characterized by feelings of (emotional, physical, and cognitive) exhaustion due to the demands of studying and an attitude of withdrawal and detachment from one's studies [9].

Numerous studies have shown that medical students experience high levels of burnout due to a highly stressful environment, competitiveness, excessive workload, sleep deprivation, peer pressure, and many other personal, curricular, institutional, and affective factors [10]. Studies on medical students have indicated the development of stress and burnout in preclinical medical education and its progression into clinical years [11,12]. Burnout in medical students has been associated with depression, sleep deprivation, thoughts of dropping out, suicidal ideation, and substance abuse [10,13–15]. It can also mitigate cognitive capabilities such as memory, interpretation of information, and skill acquisition [16].

Furthermore, burnout of medical students can also lead to burnout after they become a doctor [17] which may result in unprofessionalism, poor quality of patient care, medical errors, suicidal

ideation, and attrition, and be a factor in substance abuse and relationship difficulties [15,18]. Many studies have found that the prevalence of burnout among medical students, residents, and physicians is as much as 50% in the US [19,20]. Around the globe prevalence rates for medical students burnout ranges from 7.0% to 75.2%, depending on country-specific factors, applied instruments, cutoff-criteria for burnout symptomatology [21]. However, burnout has not been adequately explored among the medical students of Nepal. Therefore, this cross-sectional study aims to find out the prevalence of burnout among medical students of a medical college in Nepal.

Methods & Materials

Study design and settings

A cross-sectional study was done on undergraduate medical students of Nepalese Army Institute of Health Sciences (NAIHS)-College of Medicine from 14 January to 7 March, 2021. A stratified sampling technique followed by simple random sampling was employed using students' class year as a stratum. Data was collected via Google forms with the objectives explained and the consent form was attached in the form itself.

Study sample

$n = z^2 \times (p \times q) \div e^2$

$= 1.91.96^2 \times (0.49 \times 0.51) \div 0.05^2$

$= 384$

Where,

n = Sample size

z = 1.96 at 95% CI

p = 0.49 (taken 0.49 based on 48.8% prevalence of burnout in a similar Nepalese study) [22].

q = 1-p

e = marginal error, 5%

Adjusted sample size = $n \div \{1 + (n - 1) \div \mathrm{N}\}$

$= 228$

Where, N = Target population = 560*

Therefore, the calculated sample size was 228, and considering the 5% of non-response rate the sample size taken was 239.

After calculation of sample size, a stratified sampling technique was employed using students' class year as a stratum. Students' attendance sheet of each class ye[illegible]as used as a sampling frame. First, the total sample size (i.e., 239) was proportionally allocated for each stratum, then a simple random sampling method using random number generator software was applied to obtain the study participants from each class year. This was done to address the potential selection bias and response bias (detail of procedure available as **Supplementary file 1**).

Study instrument

Maslach Burnout Inventory (MBI) is the most commonly used scale for measuring burnout. Its three factors are depersonalisation, emotional exhaustion and personal accomplishment. However, studies have shown MBI's third factor to perform weakly and have recommended using a two factor model [23]. Aptly, OLBI only focuses on the two dimensions (disengagement and exhaustion), and has both positively and negatively worded questions. Also, in the context of developing countries, OLBI is more accessible as it is free to use unlike MBI [8]. Copenhagen Burnout Inventory (CBI)'s third scale, client/patient related burnout, was not appropriate for preclinical medical students (1st and 2nd years) [24].

Hence, a self-administered questionnaire containing demographic variables and OLBI-S was used for the study (available as **Supplementary file 2.**).

The demographics contained 3 items (age, gender, and academic year). OLBI-S measures burnout in two dimensions: exhaustion & depersonalization. Both subscales consist of eight items each, out of which four are positively worded and four negatively worded [9]. We recorded the participants' responses to the items on a 4 point Likert scale ranging from 1 (strongly agree) to 4 (strongly disagree).

Analytical Strategy

Both subscales, exhaustion & depersonalization, consist of four negatively worded items that have to be reversed i.e. from 1 to 4, 2 to 3, 3 to 2 & 4 to 1 so that higher scores correspond to higher burnout.

The mean of each subscale is taken and the cutoff scores ≥ 2.25 is used for exhaustion and scores ≥ 2.1 for disengagement to classify the participants into the following groups [25]:

- Low exhaustion and low disengagement (non-burnout group)
- Low exhaustion and high disengagement (disengaged group)
- High exhaustion and low disengagement (exhausted group)
- High exhaustion and high disengagement (burnout group)

Analysis was performed using STATA version 15. Simple frequency distribution was presented appropriately in table and text. Mean score presented for each scale variable and subscale where appropriate. Binary logistic regression was carried with full-blown burnout as an outcome of interest (with 1=yes and 0=no). Age, year of study and gender were independent variables and burnout was a dependent variable. Internal consistency of scale for this study was assessed using

Cronbach's Alpha. The value of Cronbach's Alpha for 16 items of scale tested was 0.777 indicating a good level of internal consistency.

Ethical consideration

Ethical clearance was obtained from the Institutional Review Committee (IRC) of NAIHS before collecting data from participants (**Ref no: 365**). All the respondents were informed about the aim and objectives of the study by including the consent form in the questionnaire itself. The participants were aware that their participation was voluntary. Confidentiality of the participants was ensured.

Results

The calculated sample size required for the study was 228. Assuming a non-response rate of 5%, we mailed 239 students, among which 229 students completely filled the questionnaires. The mean age of the participants was 22.05 ± 1.84 years (median 22, IQR 21-23). Out of 229 respondents, 148 (64.6%) were males and 81 (35.4%) were females. Similarly, 53 (23.1%) were from 5th year, 47 (20.5%) from 4th year, 45 (19.7%) from 3rd year, 40 (17.5%) from 2nd year and 44 (19.2%) from 1st year. **Table 1** summarizes the response to the individual questions of OLBI-S.

Table 1. Response to individual questions

**Positively worded questions**	**Strongly agree n(%)**	**Agree n(%)**	**Disagree n(%)**	**Strongly disagree n(%)**	**Sum [Mean ±SD]**
I always find new and interesting aspects in my studies.	47(20.5%)	159(69.4%)	16(7%)	7(3.1%)	1.926±0.6274
I find my studies to be a positive challenge	68(29.7%)	148(64.6%)	11(4.8%)	2(0.9%)	1.769±0.572
I feel more and more engaged in my studies.	24(10.5%)	131(57.2%)	69(30.1%)	5(2.2%)	2.240±0.6616
This is the only field of study that I can imagine myself doing	36(15.7%)	90(39.3%)	87(38.0%)	16(7.0%)	2.362±0.8296

I can usually manage my study-related workload well.	21(9.2%)	123(53.7%)	72(31.4%)	13(5.7%)	2.336±0.7228
I can tolerate the pressure of my studies very well.	24(10.5%)	141(61.6%)	57(24.9%)	7(3.1%)	2.205±0.6601
After a class or after studying, I have enough energy for my leisure activities.	19(8.3%)	101(44.1%)	93(40.6%)	16(7.0%)	2.463±0.7461
When I study, I usually feel energized.	24(10.5%)	138(60.3%)	62(27.1%)	5(2.2%)	2.210±0.648
**Negatively worded questions**	**Strongly agree n(%)**	**Agree n(%)**	**Disagree n(%)**	**Strongly disagree n(%)**	**Sum [Mean ±SD]**
Over time, one can become disconnected from this type of study	26(11.4%)	141(61.6%)	55(24.0%)	7(3.1%)	2.812±0.6653
Lately, I tend to think less about my academic tasks and do them almost mechanically.	10(4.4%)	117(51.1%)	83(36.2%)	19(8.3%)	2.515±0.7108
It happens more and more often that I talk about my studies in a negative way	7(3.1%)	66(28.8%)	128(55.9%)	28(12.2%)	2.227±0.6952
Sometimes I feel sickened by my studies.	11(4.8%)	108(47.2%)	82(35.8%)	28(12.2%)	2.445±0.768
There are days when I feel tired before I arrive in class or start studying.	46(20.1%)	136(59.4%)	38(16.6%)	9(3.9%)	2.956±0.7242
After a class or after studying, I tend to need more time than in the past in order to relax and feel better.	33(14.4%)	98(42.8%)	88(38.4%)	10(4.4%)	2.672±0.7736
While studying, I often feel emotionally drained.	21(9.2%)	102(44.5%)	89(38.9%)	17(7.4%)	2.555±0.7625
After a class or after studying, I usually feel worn out and weary.	21(9.2%)	118(51.5%)	75(32.8%)	15(6.6%)	2.633±0.7410

The mean scoring under disengagement subscale was 2.287±0.349 (Median: 2.375; IQR: 2.125-2.500) and the mean scoring under exhaustion subscale was 2.504±0.428 (Median: 2.500; IQR: 2.250-2.813). Likewise, the mean of total score under disengagement subscale was 18.297±2.7955 (Median: 19; IQR: 17-20) and the mean scoring under exhaustion subscale was 20.031±3.4264 (Median: 20; IQR: 18-22.5).

In accordance with the previous mentioned cutoff values, 151 (65.9%) respondents fell under burnout group, 26 (11.4%) fell under disengaged group, 29 (12.7%) fell under exhausted group whereas 23 (10.0%) fell under non-burnout group. **Fig 1** summarizes the burnout categories of the participants.

Fig 1. Burnout categories among participated medical students

Binary logistics regression for outcome burnout did not differ across age in years, gender and year of study. **Table 2** summarizes the Binary logistic regressions for occurrence of burnout.

Table 2. Binary logistic regression for occurrence of burnout

Burnout outcome	Adjusted OR	Std. Err.	z	P>|z|	[95% Conf. Interval]	
Age years	1.176098	.1692867	1.13	0.260	.8869971	1.559426
Gender						
Female®						
Male	.983714	.3024495	-0.05	0.957	.5384696	1.797118
Year of study						
3rd year®						
1st Year	1.310118	.7101023	0.50	0.618	.4528427	3.7903
2nd Year	.8037116	.3911503	-0.45	0.653	.3096261	2.086234
4th Year	.8798762	.4321055	-0.26	0.794	.3360456	2.303801
5th Year	.3545889	.1848251	-1.99	0.047	.1276576	.9849255
Constant	.0718285	.2233576	-0.85	0.397	.000162	31.8558

Discussion

Our study aimed to find the prevalence of burnout among undergraduate medical students and its association with the demographic factors such as age, sex and year of study. The prevalence of burnout among undergraduate medical students in our study is 65.9%, which is similar to the previous studies [12,26,27]. In several studies conducted in the US, the prevalence of burnout was

found to be as much as 50% [19,20]. However, according to Frajerman et al., the worldwide burnout prevalence for medical students lies around 44% [28]. And, the burnout calculated using Oldenburg Burnout Inventory (OLBI), ranged between 47.0 and 53.0% [21].

There is a considerable degree of heterogeneity in the prevalence of burnout among medical students. This is due to use of different scales for measuring burnout and also differences in criteria for categorizing burnout within the same scale. In addition to this, the divergence in the prevalence may also be due to multitude of factors like difference in curriculum, teaching methods, available facilities and other factors.

Our study found out the prevalence of burnout to be considerably higher than the study conducted prior in Nepal which reported the prevalence to be 48.8% [22]. This disparity in result could be due to the different instruments (CBI & OLBI) used to measure burnout. The higher prevalence in our study may also be due to timing of the study period, which was a month after classes resumed post-COVID-19 lockdown. Still multiple factors of the ongoing pandemic may have varying effects on the prevalence of burnout for different individuals. Furthermore, the higher prevalence of burnout may be due to the forthcoming final board examinations.

Regarding demographic data, there is no significant association of gender with the burnout in our study which is similar to the findings of some prior studies [29–31] but contrary to the studies which show high burnout in females compared to males [12,32]. Also, no significant association was found between different age groups of students, which is similar/contrary to the findings of previous studies [18,33].

The prevalence of burnout in our study was found to be 70.5% in 1st year, 62.5% in 2nd year, 71.1% in 3rd year, 72.3% in 4th and 54.7% in 5th year. Some prior studies have also shown an increasing trend in the prevalence of burnout throughout their medical studies [34–36]. But we

didn't find any correlation between burnout and year of study. This may signify the hectic schedule, competitiveness, highly stressful environment, excessive workload, sleep deprivation, peer pressure, and many other personal, curricular, institutional, and affective factors throughout the years of medical education.

Among the preclinical year students (1st and 2nd years), prevalence of burnout was found to be higher in the first year than in the second. The first year medical students have a high level of stress even in the stage prior to admission to medical school because many of them spend several years getting accepted to medical school. Also after admission, coping up with an entirely new environment away from home, living on their own, competitive spirits, lack of leisure time or activities can be various contributory factors for high burnout in first year medical students.

Among the clinical year students (3rd, 4th and 5th years), the prevalence of burnout among fifth year students was relatively lower, yet insignificant, than the third and fourth year students. This is contrary to the expected result of higher prevalence of burnout among final year students due to their heavier curriculum and examinations load. However, only a longitudinal study of the same batch of students throughout their 1st to 5th year of study would give a clearer picture of the trend of burnout in different academic years. Final year students have to appear for examinations of the major subjects (Internal medicine, Surgery, Obstetrics & Gynecology and Pediatrics) to which they already have exposure to since the start of their clinical years (third year). Despite the academic load, they have been acquainted with the examination pattern and could be less anxious about it.

Some limit[illegible]ns of the study are acknowledged. The study was conducted using an online self-reporting questionnaire, yet we had a good response rate of 95.8% (n=229). Although the selection and response bias was minimized, the social desirability bias might have affected the results of our

study. The level of burnout among the academic years may be affected by the final board examinations that are held at different times for the respective year. The study period was a month after classes resumed post-COVID-19 lockdown. However, multiple factors of the ongoing pandemic may have varying effects on the level of burnout for different individuals.

Finally, the results may not be representative of all the medical colleges of Nepal since the participants were only from NAIHS but it will surely start the much needed conversation on this topic. Furthermore, the role of factors such as gender, age, academic year, extracurricular activities, hosteller, relationship status, payment/scholarship students, sleep deprivation, etc. on burnout need further exploration.

Conclusion

The prevalence of burnout among medical students in our study was much higher than expected, and no associations of burnout with age, gender or year of study were found. Hence, effective strategies for mental well-being of future physicians need to be made by relevant authorities such as psychological help desk, mental health support groups and amendments in the academic curriculum.

Declarations

Ethics approval and consent to participate Explained consent taken before participation.

Consent for publication Not applicable

Availability of data and materials the data analyzed during the current study are available in the manuscript.

Competing interests, the authors declare that they have no competing interests.

Funding This article did not receive any specific grant from funding agencies in the public, commercial, or any other sectors.

Authors' contributions: DBS, NK, and UB contributed to the concept and design. DBS performed statistical analysis. DBS and NK guided and supervised the data collection and drafting. AT, AP, AG, MS, UB, and PB contributed to the literature search, data collection, and initial manuscript drafting. DBS, NK and PB revised the manuscript for important intellectual content and approval of the final manuscript.

All authors were involved in drafting and revising the manuscript and approved the final version.

Authors' information

DBS Medical officer, Department of Emergency Medicine, Mangalbare Hospital, Morang, Nepal

Email: medhan75@gmail.com

ORCid: 0000-0002-8121-083X

NK Consultant Neuro-psychiatrist, Department of Psychiatry, Nepalese Army Institute of Health Sciences (NAIHS), Shree Birendra Hospital, Kathmandu, Nepal

Email: katuwalnagen@gmail.com

AT Medical Student, Nepalese Army Institute of Health Sciences (NAIHS), College of Medicine, Kathmandu, Nepal

Email: a.tmng@outlook.com

ORCid: 0000-0002-3545-4841

AP Medical Student, Nepalese Army Institute of Health Sciences (NAIHS), College of Medicine, Kathmandu, Nepal

Email: agrimapaudel14@gmail.com

ORCid: 0000-0001-7556-1273

AG Medical Student, Nepalese Army Institute of Health Sciences (NAIHS), College of Medicine, Kathmandu, Nepal

Email: unagautam44@gmail.com

ORCid: 0000-0001-6277-8940

MS Medical Student, Nepalese Army Institute of Health Sciences (NAIHS), College of Medicine, Kathmandu, Nepal

Email: smuna2055@gmail.com

ORCid: 0000-0001-5088-4404

UB Medical Student, Nepalese Army Institute of Health Sciences (NAIHS), College of Medicine, Kathmandu, Nepal

Email: ujwalbhusal1@gmail.com

ORCid: 0000-0003-4741-0528

PB Medical officer, Dr Iwamura Memorial Hospital, Bhaktapur, Nepal

Email: pravash.budhathoki123@gmail.com

ORCid: 0000-0001-8856-5417

References

1. Maslach C, Jackson SE. The measurement of experienced burnout. J Organ Behav. 1981;2: 99–113. doi:10.1002/job.4030020205

2. Demerouti E, Bakker AB, Nachreiner F, Schaufeli WB. The job demands-resources model of burnout. J Appl Psychol. 2001;86: 499–512. doi:10.1037/0021-9010.86.3.499

3. Leiter MP, Schaufeli WB. Consistency of the burnout construct across occupations. Anxiety, Stress Coping. 1996;9: 229–243. doi:10.1080/10615809608249404

4. Maslach C, Leiter MP, Schaufeli W. Measuring Burnout. Oxford University Press; 2008. doi:10.1093/oxfordhb/9780199211913.003.0005

5. McCarthy ME, Pretty GM, Catano V. Psychological sense of community and student burnout. J Coll Stud Dev. 1990;31: 211–216.

6. Schaufeli WB, Martínez IM, Pinto AM, Salanova M, Bakker AB. Burnout and Engagement in University Students. J Cross Cult Psychol. 2002;33: 464–481. doi:10.1177/0022022102033005003

7. Demerouti E, Demerouti E, Bakker AB, Vardakou I, Kantas A. The Convergent Validity of Two Burnout Instruments. Eur J Psychol Assess. 2003;19: 12–23. doi:10.1027//1015-5759.19.1.12

8. Demerouti E. The Oldenburg Burnout Inventory: A good alternative to measure burnout and engagement. Handb Stress Burn Heal Care. 2008.

9. Reis D, Xanthopoulou D, Tsaousis I. Measuring job and academic burnout with the Oldenburg Burnout Inventory (OLBI): Factorial invariance across samples and countries.

Burn Res. 2015;2: 8–18. doi:10.1016/j.burn.2014.11.001

10. Pacheco JP, Giacomin HT, Tam WW, Ribeiro TB, Arab C, Bezerra IM, et al. Mental health problems among medical students in Brazil: a systematic review and meta-analysis. Rev Bras Psiquiatr. 2017;39: 369–378. doi:10.1590/1516-4446-2017-2223

11. Ashkar K, Romani M, Musharrafieh U, Chaaya M. Prevalence of burnout syndrome among medical residents: experience of a developing country. Postgrad Med J. 2010;86: 266 LP – 271. doi:10.1136/pgmj.2009.092106

12. Fares J, Saadeddin Z, Al Tabosh H, Aridi H, El Mouhayyar C, Koleilat MK, et al. Extracurricular activities associated with stress and burnout in preclinical medical students. J Epidemiol Glob Health. 2015;6: 177. doi:10.1016/j.jegh.2015.10.003

13. Youssef FF. Medical Student Stress, Burnout and Depression in Trinidad and Tobago. Acad Psychiatry. 2016;40: 69–75. doi:10.1007/s40596-015-0468-9

14. Mazurkiewicz R, Korenstein D, Fallar R, Ripp J. The prevalence and correlations of medical student burnout in the pre-clinical years: A cross-sectional study. Psychol Health Med. 2012;17: 188–195. doi:10.1080/13548506.2011.597770

15. Dyrbye L, Shanafelt T. A narrative review on burnout experienced by medical students and residents. Med Educ. 2016;50: 132–149. doi:10.1111/medu.12927

16. Miranda-Ackerman RC, Barbosa-Camacho FJ, Sander-Möller MJ, Buenrostro-Jiménez AD, Mares-País R, Cortes-Flores AO, et al. Burnout syndrome prevalence during internship in public and private hospitals: a survey study in Mexico. Med Educ Online. 2019;24.

doi:10.1080/10872981.2019.1593785

17. Dyrbye LN, Thomas MR, Huntington JL, Lawson KL, Novotny PJ, Sloan JA, et al. Personal Life Events and Medical Student Burnout: A Multicenter Study. Acad Med. 2006;81: 374–384. doi:10.1097/00001888-200604000-00010

18. Dyrbye LN, West CP, Satele D, Boone S, Tan L, Sloan J, et al. Burnout Among U.S. Medical Students, Residents, and Early Career Physicians Relative to the General U.S. Population. Acad Med. 2014;89: 443–451. doi:10.1097/ACM.0000000000000134

19. Chang E, Eddins-Folensbee F, Coverdale J. Survey of the Prevalence of Burnout, Stress, Depression, and the Use of Supports by Medical Students at One School. Acad Psychiatry. 2012;36: 177. doi:10.1176/appi.ap.11040079

20. Shanafelt TD, Boone S, Tan L, Dyrbye LN, Sotile W, Satele D, et al. Burnout and Satisfaction With Work-Life Balance Among US Physicians Relative to the General US Population. Arch Intern Med. 2012;172: 1377. doi:10.1001/archinternmed.2012.3199

21. Erschens R, Keifenheim KE, Herrmann-Werner A, Loda T, Schwille-Kiuntke J, Bugaj TJ, et al. Professional burnout among medical students: Systematic literature review and meta-analysis. Med Teach. 2019;41: 172–183. doi:10.1080/0142159X.2018.1457213

22. Pokhrel NB, Khadayat R, Tulachan P. Depression, anxiety, and burnout among medical students and residents of a medical school in Nepal: A cross-sectional study. BMC Psychiatry. 2020;20. doi:10.1186/s12888-020-02645-6

23. Kalliath T, O'Driscoll M, Gillespie D, Bluedorn A. A test of the Maslach Burnout Inventory

in three samples of health professionals. Work Stress. 2000;14: 35–50. doi:10.1080/026783700417212

24. Kristensen TS, Borritz M, Villadsen E, Christensen KB. The Copenhagen Burnout Inventory: A new tool for the assessment of burnout. Work Stress. 2005;19: 192–207. doi:10.1080/02678370500297720

25. Peterson U, Demerouti E, Bergström G, Åsberg M, Nygren Å. Work characteristics and sickness absence in burnout and nonburnout groups: A study of Swedish health care workers. Int J Stress Manag. 2008;15: 153–172. doi:10.1037/1072-5245.15.2.153

26. Almalki SA, Almojali AI, Alothman AS, Masuadi EM, Alaqeel MK. Burnout and its association with extracurricular activities among medical students in Saudi Arabia. Int J Med Educ. 2017;8: 144–150. doi:10.5116/ijme.58e3.ca8a

27. Dyrbye LN, Power D V, Massie FS, Eacker A, Harper W, Thomas MR, et al. Factors associated with resilience to and recovery from burnout: a prospective, multi-institutional study of US medical students. Med Educ. 2010;44: 1016–1026. doi:10.1111/j.1365-2923.2010.03754.x

28. Frajerman A, Morvan Y, Krebs M-O, Gorwood P, Chaumette B. Burnout in medical students before residency: A systematic review and meta-analysis. Eur Psychiatry. 2020/01/01. 2019;55: 36–42. doi:DOI: 10.1016/j.eurpsy.2018.08.006

29. Backović D V, Zivojinović JI, Maksimović J, Maksimović M. Gender differences in academic stress and burnout among medical students in final years of education. Psychiatr Danub. 2012;24: 175–181.

30. Al-Jehani YM, Althwanay AM, Buainain HM, Abuhaimed AK, Almulhim AM, Abusrir FA, et al. Burnout Prevalence and Associated Stressors in Medical Students of Traditional and Problem-Based Learning Curricula in a Saudi University. Saudi J Med Med Sci. 8: 125–132. doi:10.4103/sjmms.sjmms_301_19

31. Bore M, Kelly B, Nair B. Potential predictors of psychological distress and well-being in medical students: a cross-sectional pilot study. Adv Med Educ Pract. 2016;7: 125–35. doi:10.2147/AMEP.S96802

32. Thun-Hohenstein L, Höbinger-Ablasser C, Geyerhofer S, Lampert K, Schreuer M, Fritz C. Burnout in medical students. neuropsychiatrie. 2021;35: 17–27. doi:10.1007/s40211-020-00359-5

33. O’Connor K, Muller Neff D, Pitman S. Burnout in mental health professionals: A systematic review and meta-analysis of prevalence and determinants. Eur Psychiatry. 2018;53: 74–99. doi:10.1016/j.eurpsy.2018.06.003

34. Voltmer E, Rosta J, Aasland OG, Spahn C. Study-related health and behavior patterns of medical students: A longitudinal study. Med Teach. 2010;32: e422–e428. doi:10.3109/0142159X.2010.496008

35. Nteveros A, Kyprianou M, Artemiadis A, Charalampous A, Christoforaki K, Cheilidis S, et al. Burnout among medical students in Cyprus: A cross-sectional study. PLoS One. 2020;15: e0241335. doi:10.1371/journal.pone.0241335

36. Fitzpatrick O, Biesma R, Conroy RM, McGarvey A. Prevalence and relationship between burnout and depression in our future doctors: a cross-sectional study in a cohort of

preclinical and clinical medical students in Ireland. BMJ Open. 2019;9: e023297. doi:10.1136/bmjopen-2018-023297

Figure legends

Fig 1. Burnout categories among participated medical students

 Click here to access/download;Figure;Fig 1..tif

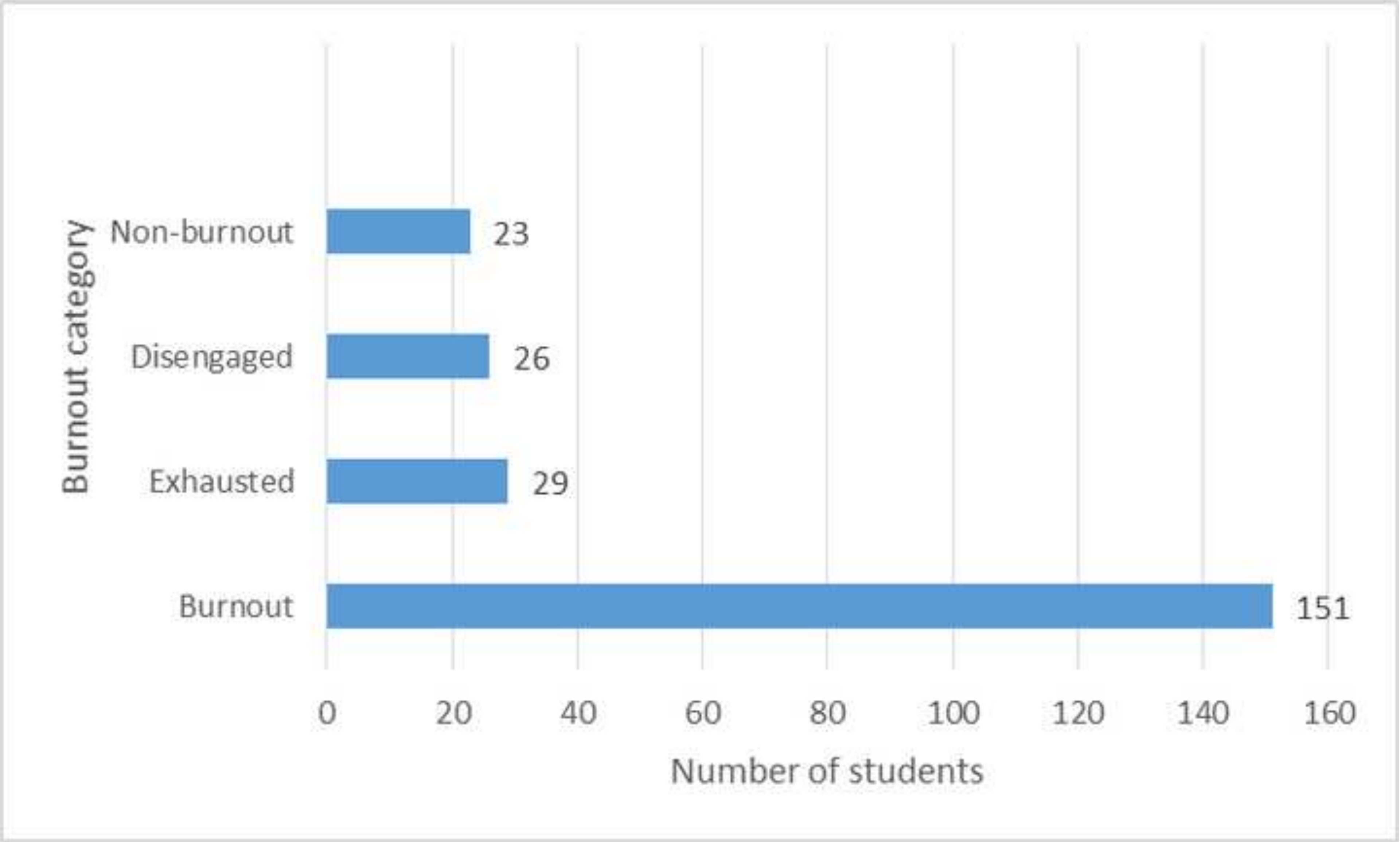

Click here to access/download
Supporting Information
Supplementary file 1. Corrected IRC Proposal.pdf

Click here to access/download
Supporting Information
Supplementary file 2. OLBI Questionnaire.pdf

Click here to access/download
Supporting Information
Supplementary file 3. Burnout.dta
