# Supplementary material for: Burnout among medical students of a medical college in Kathmandu; A cross-sectional study
Source: PLoS One. 2021 Jun 24;16(6):e0253808. doi: 10.1371/journal.pone.0253808 (PMC8224915; doi:10.1371/journal.pone.0253808)
Supplement: Attachment — Submitted filename: Review of Burnout among medical students of a medical college in Kathmandu_A cross sectional study.docx [file pone.0253808.s004.docx]

Review of

Burnout among medical students of a medical college in Kathmandu: A cross sectional study

1. **Reviewer’s Overall Comment:**

Research on burnout and emotional exhaustion in Nepal for medical professionals and students is increasing. Nevertheless, there is a gap in the study of burnout among preclinical students. The relevancy of the research is well articulated and explained in the Nepali context. This research, scientifically designed and meticulously carried out after following exact research methods, will support the education and medical institutions to look at the burden of medical education on their students. With minor revisions to be addressed, the reviewer recommends publishing this article.

2. **Few comments for adjustment:**

- Line 31 – please be specific that the researchers had used the English version of the Oldenburg Burnout Inventory adapted for students. It seems that the tool was not translated and culturally adapted in the research process. Replicate the same in line 105.
- In abstract's result section, line 33, please include the total number of respondents (N = 229).
- Line 78, please describe the sampling process and cite the formula you have used to define the sample size for this research. It would be nice to explain, the reasons for choosing the sample size in that way before straightforwardly putting the formula.
- The data were collected using a google form, was there any record of individual data such as the email of the respondents? Did the participants receive any debriefing note with an explanation of the burnout including exhaustion and disengagement? Did the participant receive any guidance to seek support if they wish?
- Line 136 – how do you explain that the confidentiality and anonymity of the participants were ensured? Kindly explain the process from data collection to data analysis and repository phase of the data.
- Line 173, please mention (N = 651) after % of prevalence and response rate of their study.
- After line 197, it would be worth mentioning that the peer and family pressure to be a medical doctor in Nepali society creates pressure on the students.
- Pokhrel, Khadayat, and Tulachan (2020) outlined other possible causes of higher prevalence of burnout in Medical students – *‘the field of medicine not being one’s first priority, following friends to study medicine, parental wish to study medicine, wrongly choosing the specialty, and being demotivated after learning the reality of medicine. The authors, unfortunately, came across a few circumstances in which some medical students became demotivated after living the reality of medicine. Most of them followed their parents’ wish or dreamt of their higher social status before enrolling into the medical school (p.15).’*

The reviewer recommends highlighting such findings in the discussion section.

- Authors have suggested some of the strategies and actions to address the burnout of medical students in the conclusion section. Line 223-225 says, *‘Hence, effective strategies for the mental well-being of future physicians need to be made by relevant authorities such as psychological help desk, mental health support groups, and amendments in the academic curriculum’*. It is understandable that this study limits after knowing the prevalence of burnout, nevertheless, it would be great to review secondary literature on effective and efficacious actions for the prevention of burnout in medical students and describe it in the discussion section for future researchers and academia leaders. Here are some of the resources available to similar populations to address burnout of medical students:
- Adhikari (2018) on Compassion fatigue into the Nepali counselors: challenges and recommendations
- Recommendation section of Adhikari (2020) pages – 131 to 134,
- Equally, the study of Adhikari et al. (2017 p.6) mentions, ‘*Various solutions to reduce stress like creating a nurturing learning environment, identifying and assisting students, teaching skills for stress management and promoting self-awareness have been proposed. Integration of such programs as a part of medical education with special focus on preclinical students and female students may improve mental health among students’.*

**References**

Pokhrel, N. B., Khadayat, R., & Tulachan, P. (2020). Depression, anxiety, and burnout among medical students and residents of a medical school in Nepal: a cross-sectional study. *BMC psychiatry*, *20*(1), 1-18. <https://doi.org/10.1186/s12888-020-02645-6>

Adhikari, A., Dutta, A., Sapkota, S., Chapagain, A., Aryal, A., & Pradhan, A. (2017). Prevalence of poor mental health among medical students in Nepal: a cross-sectional study. *BMC medical education*, *17*(1), 1-7. <https://doi.org/10.1186/s12909-017-1083-0>

Adhikari, Y. (2020). Prevalence of Professional Quality of Life (ProQOL) and Its Influence on the Personal Distress of Doctors in Nepal. (Doctor of Philosophy Dissertation, University of Nicosia). <https://www.researchgate.net/publication/344397792_Prevalence_of_Professional_Quality_of_Life_ProQOL_and_Its_Influence_on_the_Personal_Distress_of_Doctors_in_Nepal>

Adhikari, Y. (2018). Compassion fatigue into the Nepali counselors: Challenges and recommendations. *MOJ Public Health*, *7*(6), 376–379. http://dx.doi.org/[10.15406/mojph.2018.07.00271](https://doi.org/10.15406/mojph.2018.07.00271)
