# Supplementary material for: Burnout among medical students of a medical college in Kathmandu; A cross-sectional study
Source: PLoS One. 2021 Jun 24;16(6):e0253808. doi: 10.1371/journal.pone.0253808 (PMC8224915; doi:10.1371/journal.pone.0253808)
Supplement: Attachment — Submitted filename: Summary of Comments.docx [file pone.0253808.s006.docx]

**Comments**

**Methodology Part**

1. How you randomly select the participants, please clearly mentioned.
2. How you make reach the form to the study participants please clearly mentioned
3. Mention total number of students
4. Properly mention inclusion and exclusion criteria
5. How do you insured validity and reliability of data as you have collected data via Google form

**Discussion Part**

1. Rewrite discussion as per journal rules and regulation. Provide references wherever other studies is included.
2. You have mentioned several limitations ,despite of those limitation how your study will play a vital role in understanding the burnout among medical students,what might be the factors that hinder burden?

**Conclusion and Recommendation Part**

1. How you can conclude that the burden is higher, do you have any comparison study.
2. Please provide a specific recommendation from student side and national medical council level?
3. What are your further recommendations to conduct the study to make it more genaralized?

**Overall Manuscript Part**

1. Improve the quality of English and proper describe the result part.
