# Supplementary material for: Burnout among medical students of a medical college in Kathmandu; A cross-sectional study
Source: PLoS One. 2021 Jun 24;16(6):e0253808. doi: 10.1371/journal.pone.0253808 (PMC8224915; doi:10.1371/journal.pone.0253808)
Supplement: Attachment — Submitted filename: Response to Reviewers PLOS.docx [file pone.0253808.s008.docx]

Joerg Heber, PhD

Editor in Chief,

PLOS ONE

San Francisco, California, US

Dear Dr. Heber,

Referring to our study “**PONE-D-21-09730: Burnout among medical students of a medical college in Kathmandu; A cross-sectional study**”, thank you for providing such an opportunity to revise and edit our manuscript to make it more clear and understandable to the audience. We author believe, after these revisions, now our manuscript is more clear and replicable to enthusiasts and researchers of such title. And, we believe this will help to modify curricula and help policymakers to review policy/curricula for medical students and health personals to decrease burnout and ease their day-to-day life.

We have revised our manuscript as per the comments by reviewers and here we have responded to every comment by editor and reviewer in a point-to-point fashion. We have mentioned replies to every comment as appropriate.

**Journal Requirements:**

Please review your reference list to ensure that it is complete and correct. If you have cited papers that have been retracted, please include the rationale for doing so in the manuscript text, or remove these references and replace them with relevant current references. Any changes to the reference list should be mentioned in the rebuttal letter that accompanies your revised manuscript. If you need to cite a retracted article, indicate the article’s retracted status in the References list and also include a citation and full reference for the retraction notice.

**Reply:** For easy handling of references we use the automatic Mendeley plugin of PLOS ONE. So, we assume the referencing style is as per journal requirements.

**Additional Academic Editor Comments:**

- Comments by Reviewer #4 have not been completely addressed in the revised manuscript. Kindly check the attachments for detailed comments made by Reviewer #4 and provide a specific point-to-point response.

**Reply:** Thank you for noting the comment. We have already amended that comment in the manuscript. But pardon us for not keeping whole changes in response. We did not find any new comments to amend further. Please find a specific response as it is done in the manuscript in the reply section of the specific comment.

Reviewer 4:

Authors are more experience of research, Article is current more relevant and Ethical approval letter and Participants consent was obtained. provide a specific recommendation from student side and national medical council level.

Reply: As pointed out by the reviewer, we have added specific recommendations from both the student side as well as national medical council level. It has been added and described already in previously revised submission in later part of discussion section as “At an individual level, students should be encouraged to incorporate various self-care techniques into their daily lives such as a nutritious diet, regular exercise, a restful sleep, making a balance between study and leisure, the practice of self-compassion, and being aware of their emotional needs [40]. Similarly, positive coping and hobbies help to overcome the stressors of medical studies. Some common practices can be mindfulness, yoga, listening to music, reading books, and outdoor games requiring group engagement [41].

Various measures can be taken by medical institutions and governing bodies like Nepal Medical Association (NMA) and Nepal Medical Council (NMC). They should create a nurturing, learning environment, teach skills for stress management and promote self-awareness. Adjustment in the curriculum and teaching methods can be made to reduce distress among medical students [42]. A trustworthy and stigma-free environment should be built for medical students and health care professionals and encouraged to seek professional counseling or therapy when in need. Further studies are required to understand distress among medical students and health care professionals, and evidence-based approaches to dealing with such difficulties. Institutions such as the University Grants Commission (UGC) and Nepal Health Research Council (NHRC) should make provisions to fund such future studies. It is also recommended to adapt and validate screening tools for use in the Nepali population in the future [43].”

We did not notice any new comments for more action. If any corrections are warranted, will be happy to amend them.

Thank you
